# High Counts and Anthracene Degradation Ability of *Streptococcus mutans* and *Veillonella parvula* Isolated From the Oral Cavity of Cigarette Smokers and Non-smokers

**DOI:** 10.3389/fmicb.2021.661509

**Published:** 2021-06-28

**Authors:** Hams A. Moussa, Reham Wasfi, Nourtan F. Abdeltawab, Salwa A. Megahed

**Affiliations:** ^1^Department of Microbiology and Immunology, Faculty of Pharmacy, October University for Modern Sciences and Arts (MSA), Giza, Egypt; ^2^Department of Microbiology and Immunology, Faculty of Pharmacy, Cairo University, Cairo, Egypt

**Keywords:** oral microbiota, smoking, polycyclic aromatic hydrocarbons biodegradation, microbial interactions, anthracene

## Abstract

The composition and metabolic functions of oral microbiota are affected by many factors including smoking leading to several health problems. Cigarette smoking is associated with changes in oral microbiota composition and function. However, it is not known if the depletion of certain bacterial genera and species is due to specific toxins in cigarette smoke, or indirectly due to competition for colonization with smoking-enriched bacteria. Therefore, the aim of this study was to determine the effect of cigarette smoking on the microbial prevalence and polycyclic aromatic hydrocarbons (PAHs) biodegradation of selected enriched and depleted oral bacteria from oral microbiota of smokers compared to that in non-smokers. Samples of oral rinse from smokers and non-smokers were collected (*n* = 23, 12 smokers and 11 non-smokers) and screened for oral bacterial strains of *Streptococcus mutans, Lactobacillus* spp., and *Veillonella* spp. Comparing counts, *S. mutans*, *V. tobetsuensis*, and *V. dispar* showed higher counts in smokers compared to non-smokers while the *Lactobacillus* spp. were higher in non-smokers. *Lactobacillus fermentum* was prevalent in smokers, representing 91.67% of the total Lactobacillus spp. isolates. The biodegradation potential of anthracene; a representative of PAHs of collected isolates, in single and mixed cultures, was assayed with anthracene as the sole source of carbon using 2,6-dichlorophenol indophenol (2,6-DCPIP) as indicator. *S. mutans* isolates recovered from smokers showed higher degradation of anthracene compared to those recovered from non-smokers. The anaerobic anthracene biodegradation activity of *V. parvula* isolates from non-smokers was the highest among all isolates of the three recovered genera from the same subject. The anthracene biodegradation potential of *Lactobacillus* spp. was variable. Combinations of isolated bacteria in co-cultures showed that *Lactobacillus* spp. interfered with anthracene biodegradation ability along with the viable counts of *S. mutans* and *Veillonella* spp. In conclusion, oral dysbiosis due to cigarette smoking was observed not only due to changes in oral bacterial relative abundance but also extended to bacterial functions such as anthracene biodegradation tested in this study. Microbe–microbe interactions changed the anthracene biodegradation potential and growth of the microbial mixture compared to their corresponding single isolates, and these changes differ according to the constituting bacteria.

## Introduction

Microorganisms particularly bacteria heavily colonize the human mouth. It has been shown that this microbial community in the human mouth is the second most complex in the body after that of the colon with over 600 different types of microbes present in the human mouth (Dewhirst et al., 2010). The bacterial community of the mouth is dominated by five phyla Firmicutes, Bacteroidetes, Proteobacteria, Actinobacteria and Fusobacteria. Members of these bacterial phyla are involved in a wide variety of functions, and many are important in maintaining oral and systemic health (Dewhirst et al., 2010). Firmicutes constitutes 33.2% of oral microbiota, and it is mainly composed of three major classes, Bacilli, Erysipelotrichia, and Clostridia (Dewhirst et al., 2010). From the Bacilli, *Streptococcus* genus has the most dominant bacterial species in the oral cavity representing 19.2% of the total oral microbiota (Al-Mudallal et al., 2008). Oral *Streptococci* produce adhesive molecules allowing them to successfully colonize different sites in the mouth. *S. mutans* is among the most acidiuric oral *Streptococcus* species leading to excessive acidification of the oral environment and it is directly associated with the occurrence of dental caries (Abranches et al., 2018). *Veillonella* genus (Class Clostridia) is gram negative strict anaerobic cocci and it is one of the most common oral bacterial species that represents 8.6% of the total oral microbiota. *Veillonella* spp. has been reported to play a significant role as early colonizers in biofilm formation and in facilitating the biofilm and dental plaque formation by other species. Members of *Lactobacillus* (Class Erysipelotrichia) are diverse gram-positive bacilli that are aerotolerant anaerobes. *Lactobacillus* has been reported as one of the most common oral species linked to dental caries due to their ability to ferment a wide range of carbohydrates and to survive in low pH (Badet and Thebaud, 2008).

Disturbance of oral microbiota are associated with periodontitis and dental caries. Moreover, there is increasing evidence of a role for oral microbiota disturbance in systemic diseases of the lungs, digestive tract and cardiovascular system (Belda-Ferre et al., 2012). There are many factors that lead to oral dysbiosis such as environmental exposures through diet, living conditions and habits that could favor the growth of a particular microbial phyla or genera in the oral cavity. One particular habit is tobacco smoking, as cigarette smoke creates an environment favoring strict or facultative anaerobes. This may result in the loss of beneficial bacterial species leading to pathogen colonization and eventually to disease. Other deleterious effects of cigarette smoke are increased acidity of saliva, alteration in bacterial adherence to mucosal surfaces and impairment of host immunity (Bizzarro et al., 2013; Kato et al., 2016; Yu et al., 2017; Stewart et al., 2018; Vallès et al., 2018; Huang and Shi, 2019). Tobacco smoke is classified as potential carcinogen for oral cavity and pharynx by the International Agency of Research in Cancer (IARC) (IARC, 2020). Tobacco smoke contains over 7,000 different chemicals including polycyclic aromatic hydrocarbons (PAHs) (Talhout et al., 2011).

PAHs are products of incomplete combustion and are known carcinogenic chemicals postulated to play a potential role in cigarette smoking carcinogenesis. There are sixteen PAHs in tobacco smoke classified by the U.S food and drug administration (FDA) as harmful and potentially harmful constituents (HPHCs) (Hecht, 2011; Lawal, 2017), among which anthracene is one of the important priority PAH pollutant. Owing to its simple structure, it is used as a model PAH in different studies to determine the factors affecting the bioavailability, biodegradation and rate of microbial degradation of PAHs in the environment (Karale et al., 2015). PAHs are highly lipophilic, so after human exposure through inhalation or ingestion, their bioavailability is significant and detectable levels of PAHs appear in almost all the human body internal organs (Moorthy et al., 2015). Microorganisms exhibit capabilities for PAHs biodegradation by their metabolic pathways by using these PAHs as carbon sources, they show an ecofriendly behavior to help in PAHs biodegradation by applying two modes of action; the aerobic biodegradation and the anaerobic biodegradation processes (Haritash and Kaushik, 2009).

Dysbiosis in oral microbiota associated with smoking is evident in studies comparing smokers to non-smokers at the phylum level. These studies showed that there is a significant depletion of Proteobacteria, Bacteroidetes and Fusobacteria and enrichment of Firmicutes and Actinobacteria in smokers compared to non-smokers (Wu et al., 2016; Vallès et al., 2018). At the genus level, there is an increased abundance of *Streptococcus* spp. in current smokers, they are facultative or obligate anaerobes and generally acid tolerant, which may explain their success in the smoking environment. It was also proved that even low concentration of cigarette’s nicotine significantly enhances the growth, biofilm formation and metabolic activities of cariogenic bacteria as *S. mutans*. (Huang et al., 2012). Additionally, there is an increase in the anaerobic *Veillonella* genus and Actinobacteria operational taxonomic unit (OTUs). Conversely, aerobes such as *Neisseria* and *Corynebacterium* are depleted in smokers. Interestingly, there is a depletion of certain anaerobic OTUs in smokers as well, including *V. parvula, Leptotrichia* spp., and *Peptostreptococcus* spp. It is possible that these bacteria were depleted due to specific toxicants in cigarette smoke, or indirectly due to competition for colonization with smoking enriched bacteria (Wu et al., 2016; Vallès et al., 2018). The depletion of certain xenobiotic biodegradation pathways in current smokers suggests important functional losses with potential health consequences, as the oral bacteria are first to come into contact with cigarette smoke as it enters the human body, and may play an important role in degrading the accompanying toxic compounds (Wu et al., 2016).

Although the biodegradation of PAHs by soil bacteria isolated from contaminated sites is well studied but PAHs biodegradation by human microbiota in particular the oral ones is nascent. Moreover, previous studies dealing with human microbiota and cigarette smoking were observational or in silico and did not show if the changes of oral microbial prevalence in smokers is due to direct effects of toxicants in cigarettes or due to indirect effect of oral microbe-microbe interaction. Hence the current study was carried out to understand the effect of anthracene (one of the cigarette toxicant) on selected oral bacteria and to determine the effect of anthracene on degradation potential of these bacteria and their viability either in single or mixed cultures (co-cultures) to determine the effect of microbe-microbe interaction. The selected bacteria were *S. mutans*, *Veillonella* spp. and *Lactobacillus* spp. belonging to the most dominant oral phylum Firmicutes that represents 33% of the total oral microbiota.

## Materials and Methods

### Participants

Sixteen smokers and fourteen non-smokers were enrolled in this study and provided oral rinse samples after giving their informed consent following the approval of the Research Ethics committees of the Faculty of Pharmacy at October University for Modern Sciences and Arts (MSA) with approval ID (M2/EC2/2016MS) and Faculty of Pharmacy, Cairo University with approval ID MI (1722). Participants recruited in this study were asked to fill in a questionnaire embodying information about smoking status (never/former/current smoker), tobacco consumption, smoking duration, smoking dose. All participants were chosen to be >18 years of age, and all participants with the following criteria were excluded: (1) recent antibiotic treatment or steroidal drug use (<2 weeks); (2) clinical signs of periodontal disease; (3) underlying chronic or systemic disease; (4) smoking of e-cigarettes, water pipes and all other smoking tools other than cigarettes; (5) ceased smoking; (6) pregnant or breast feeding for female participants (Ghannoum et al., 2010; Gaudet et al., 2013; Vallès et al., 2018). Smoker subjects were smoking ≥ 5 cigarettes/day for at least 1 year till time of sample collection and they were further classified according to the number of daily cigarettes consumption into light, intermediate and heavy smokers for those who consume <10, 10–20, and >20 cigarettes/day, respectively (Lohse et al., 2016).

### Standard Bacterial Strains

Standard bacterial strains used in the study were *Veillonella parvula* ATCC 10790, *V. dispar* ATCC 17748, *S. mutans* ATCC 25175, *Lactobacillus salivarius* ATCC 11741, and *Bacillus subtilus* ATCC 6051.

### Collection of Oral Rinse

For oral rinse samples’ collection, each participant was asked to refrain from eating and drinking for one hour prior to sample collection, rinse mouth for 60 s with 10 ml sterile saline, then spill saline wash into 50 ml sterile tube to be immediately transferred on ice for processing in the microbiology laboratory following the procedure of Vallès et al. (2018).

### Enumeration and Isolation of *S. mutans*, *Lactobacillus* spp., and *Veillonella* spp. From Concentrated Oral Rinse

Each oral rinse sample was then centrifuged at 1,900×g for 10 min, the supernatant was discarded and the pellets were resuspended in 2.5 ml saline to produce a concentrated homogenous suspension (Latimer et al., 2015). Bacterial suspensions were diluted 10-fold serial dilutions and aliquots were plated on custom-made media: Tryptone Yeast Extract Cystine with Sucrose and Bacitracin (TYCSB) (Wan et al., 2002), *Lactobacillus* Selective agar (LBS) (Coeuret et al., 2003) and *Veillonella* selective agar medium (Djais et al., 2017) for isolation of *S. mutans*, *Lactobacillus* spp., and *Veillonella* spp., respectively. Bacteria were incubated anaerobically at 37°C for 48 h for the enumeration of *S. mutans* (Al-Mudallal et al., 2008) and *Lactobacillus* spp., while the slowly growing Veillonella spp. required incubation for 72 h (Igarashi et al., 2009). After anaerobic incubation, colonies with characteristic appearance of each bacteria were counted and selected for further identification by Gram staining, followed by molecular identification for each species. Isolates were preserved in glycerol stock at −20°C.

### Biochemical and Molecular Identification of Bacterial Isolates

We combined multiple molecular and biochemical approaches to identify the genus and species to give more credibility to species-level identification, which usually cannot be achieved by sequencing the SSU rRNA alone. Isolated bacteria were categorized into Gram positive or negative after staining by Gram stain and examination under microscope. *S. mutans* was biochemically identified by catalase production, dextran production, sugar fermentation and tolerance to high concentration of sodium chloride. Colonies growing on selective media for *S. mutans* and *Veillonella* spp. were grown in brain heart infusion (BHI) broth while, those for *Lactobacillus* spp. were cultured in De Man, Rogosa and Sharpe (MRS) broth for 48 h under anaerobic conditions at 37°C. Genomic DNA extraction was done using GeneJET Genomic DNA Purification kit (Thermo Fisher Scientific Surrey, United Kingdom) according to the instruction of manufacturer.

*Lactobacillus* were identified by amplification and sequencing of the *16s rRNA* gene following the method of Weisburg et al. (1991) at the Macrogen sequencing facility (Macrogen, South Korea).

Identification of oral *S. mutans* was confirmed by the PCR amplification of the glucosyl transferase (*gtfB*) gene responsible for the production of extracellular glucans that play a major role in dental plaque following the method of Oho et al. (2000).

The colonies showing typical morphology on *Veillonella* selective agar were first identified at the genus level using the method of Arif et al. (2008). Species of oral *Veillonella* were identified using species-specific primers following the method of Mashima et al. (2016). All primers used in this study were synthesized by Invitrogen (Surrey, United Kingdom) and are listed in Table 1.

**TABLE 1:** Primers used for molecular identification of *Streptococcus mutans* and *Veillonella* spp.

Primer	Sequence (5′–3′)	Annealing temperature (°C)	Amplicon size (bp)	Bacteria identified (references)
*gtfB*^a^ forward *gtfB* reverse	ACTACACTTTCGGGTGGCTTGG CAGTATAAGCGCCAGTTTCATC	52	96	*Streptococcus mutans* (Oho et al., 2000)
*Veillonella rpoB^b^* forward *Veillonella rpoB* reverse	GTAACAAAGGTGTCGTTTCTCG GCACCRTCAAATACAGGTGTAGC	55	700	*Veillonella* as a genus (Mashima et al., 2016)
*Veillonella* reverse *V. parvula forward *V. dispar forward *V. denticarioasae forward	GTGTAACAAGGGAGTACGGACC GAAGCATTGGAAGCGAAAGTTTCG AACGCGTTGAAATTCGTCATGAAC GAAAGAAGCGCGCACCGACAGT	57	– 623 321 692	*V. parvula*, *V*. *dispar*, and *V. denticariosae* reactions with *Veillonella* reverse primer (Igarashi et al., 2009)
*Veillonella* forward **V. atypica reverse **V. rogosa reverse	GTAACAAAGGTGTCGTTTCTCG AGCAGCTTCTTCTACGTGACC GATCCATTTCTGGAGCATCC	55	– 386 510	*V. atypica and V. rogosae* reaction with *Veillonella* forward primer (Mashima et al., 2016)
*V. tobetsuensis* forward *V. tobetsuensis* reverse	CTCTCAACGTCAAGCAACAAAAGATGC GATAAGGTAGTTCATGATGCGTTGG	57	265	*V. tobetsuensis* (Mashima and Nakazawa, 2015)

*^a^ Glucosyl transferase B.*

^b^ RNA polymerase β subunit.

*Species-specific oligonucleotides used with primer *Veillonella* reverse.

**Species-specific oligonucleotides used with primer *Veillonella* forward.

The thermal cycler used was Biometra thermal cycler (Hamburg, Germany). PCR products were detected by agarose gel electrophoresis (1.5% w/v) using Tris Acetate EDTA (TAE) as the running buffer and gel loading buffer (Pelt-Verkuil et al., 2008).

### Determination of Anthracene Biodegradation Ability of Bacterial Isolates by 2, 6-Dichlorophenol Indophenol (2, 6-DCPIP) Colorimetric Assay

*S. mutans* and *Veillonella* spp. were grown in brain heart infusion (BHI) broth while, *Lactobacillus* spp. was cultured in MRS broth for 48 h under anaerobic conditions at 37°C. The cultures were then centrifuged at 1,900×g for 10 min and the supernatant was discarded. The pellet was washed twice with 0.9% sterile saline to remove any traces of the medium and resuspended in 0.9% sterile saline to produce a culture with turbidity equivalent to OD of 1 at 600 nm which corresponds approximately to the turbidity of a homogenous suspension of bacteria of 5 × 10^9^cfu/ml (Karale et al., 2015).

The ability of the isolates to utilize anthracene substrate can be measured by observing the disappearance of the blue color of the 2,6-DCPIP with time (Karale et al., 2015). The 2,6-DCPIP assay was carried out according to the method of Chowdhury et al. (2017) with modifications. The assay was carried out to evaluate the capability of single or combined isolates for the biodegradation of anthracene. Assay was done on individual bacterial isolates from smokers and non-smokers and also in three replicates of the respective standard strains including: *S. mutans*, *V. parvula*, *V. dispar*, and *L. salivarius*, also different combinations of bacterial isolates including different co-cultures of duos mixtures (1:1 v/v) of *S. mutans* and *Veillonella* spp., *S. mutans* and *Lactobacillus* spp., *Lactobacillus* spp., and *Veillonella* spp. and triples mixtures (1:1:1 v/v/v) of *S. mutans*, *Veillonella* spp. and *Lactobacillus* spp. The capability of the whole oral rinse (consortium) from selected smokers (S 11 and 12) and non-smokers participants (NS 10 and 11) to degrade anthracene was also tested.

A volume of 2.4 ml bacterial suspension (O.D.1 at 600 nm) was used to inoculate the assay mixture containing 22.5 ml Fe free W medium, 1.5 ml FeCl_3_ 6.H_2_O solution (150 μg/l), 1.5 ml of 2,6-DCPIP solution (50 μg/l) followed by addition of 0.25 ml of anthracene (1 mg/ml in dichloromethane). The reaction mixture was anaerobically incubated at 37°C for 8 days. Anthracene biodegradation ability was indicated by change in the color of the medium from blue to colorless (Karale et al., 2015). Three controls were simultaneously run: a positive control with *B. subtilus* (one of the highest anthracene biodegrader microorganisms), a negative control without bacterial suspension and another negative control without anthracene. Negative controls were done to assure that there is no significant spontaneous reduction in 2, 6-DCPIP color.

Aliquots from the assay mixture were pipetted at 0, 1, 2, 7, and 8 days over a period of 8 days of incubation. Aliquots were centrifuged at 7,700×g for 15 min to avoid the interference between bacterial growth turbidity and indicator’s color. The anthracene biodegradation was estimated by measuring the change in absorbance of the DCPIP indicator. Absorbance readings were converted to concentrations using a standard curve. A standard curve for DCPIP was constructed using different concentrations from 244X10^–8^ (Absorbance = 0) to 5X10^–3^ g/L (Absorbance = 2.55), with correlation coefficient (*r* = 0.9984). The equation Y = 501×−0.0071 was obtained from the curve, y represents the OD of DCPIP of each × indicates the corresponding concentration in mg/ml (Supplementary Figure 1). The percentage of remaining DCPIP color intensity was calculated by comparing the change in color intensity after a given time with the initial value based on Chowdhury et al., 2017 equation:

$$\mathrm{Percent}\,\mathrm{color}\,\mathrm{reduction}=\frac{\mathrm{Final}\,\mathrm{DCPIP}\,\mathrm{indica}\,\text{to}\,r\,\mathrm{concentration}}{\mathrm{Initial}\,\mathrm{DCPIP}\,\mathrm{indica}\,\text{to}\,r\,\mathrm{concentration}}\times 100$$

The growth of the bacteria was monitored during the assay by drop plate technique on selective medium for each genus (Esteban Florez et al., 2016).

### Statistical Analysis

Statistical analysis was performed using GraphPad Prism 5.01 (GraphPad software Inc., CA, United States). The results were presented as median and error ± interquartile range. Kruskal Wallis and multiple comparisons using Dunn’s corrections were used to compare the prevalence of *S. mutans, Lactobacillus* spp., and *Veillonella* spp. in smokers and non-smokers subjects and viable counts of the three microorganisms in anthracene biodegradation study where *p* < 0.05 was considered to be statistically significant. Data of the anthracene biodegradation by the three microorganisms and their mixtures were statistically analyzed by Mann-Whitney *U*-test and multiple comparisons using Holm Sidak’s corrections where *p* < 0.05 was also considered to be statistically significant.

## Results

### Demographic Data on Study’s Participants

Applying selection criteria mentioned in materials and methods section, 27 from the two genders out of the 30 participants were found eligible for including and handling their oral rinses’ samples in the study and 3 samples were excluded due to local oral disease (severe periodontitis) or systemic disease (diabetes). The average ages of smoker and non-smoker participants was 21–40 and 25–30 years old, respectively, and the average duration of smoking was 1–15 years. The average cigarette’s consumption was 6–60 cigarettes/day (Supplementary Table 1). The fourteen smoker participants were divided according to cigarettes consumption into heavy smokers (*n* = 5), intermediate smokers (*n* = 7) and light smokers (*n* = 2). One of the limitations of the current study was the small sample size, but it was appropriate for proper statistical testing.

### Prevalence of *S. mutans, Lactobacillus* spp., and *Veillonella* spp. in Smokers and Non-smokers Groups

**FIGURE 1:**
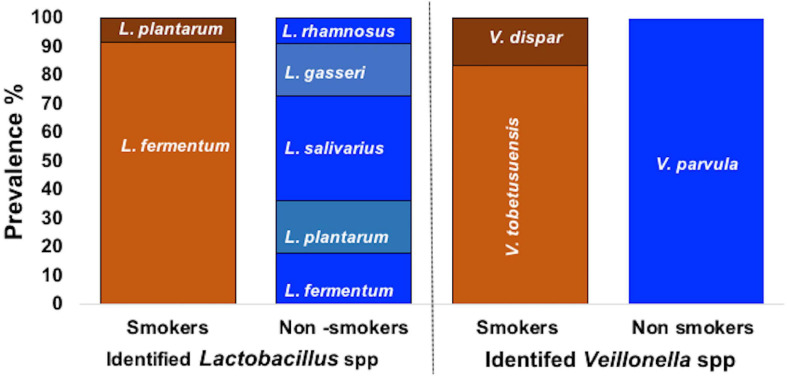
Prevalence of *Lactobacillus* spp., and *Veillonella* spp. in smokers and non-smokers groups.

From the 27 samples, 4 were neglected due to failure in isolation of the three required bacterial genus from the same participant and a total of 69 bacterial strains were isolated from 23 oral rinses. Isolates recovered on selective media were preliminary identified by microscopic examination revealing Gram positive cocci bacteria with chain arrangement, Gram negative cocci or diplococci cells arranged in masses or short chains and Gram-positive rod-shaped cells for *S. mutans*, *Veillonella* spp. and *Lactobacillus* spp., respectively. Biochemically, isolates of *S. mutans* were identified by being devoid of catalase enzyme, tolerant to 4% NaCl, their ability to produce dextran and to ferment sugars (namely: mannitol, sorbitol, sucrose, and inulin). Further molecular identification was carried out for confirmation. Molecular detection of *gtfB* gene in colonies growing on TYCSB confirmed their identification as *S. mutans* with 100% prevalence in samples obtained from all smoker and non-smokers groups (Supplementary Figure 2). Sequencing of the 16s *rRNA* gene of colonies growing on *Lactobacillus* Selective agar (LBS) was used to identify various species, and according to the similarity score defined previously by Sabat et al. (2017), bacteria were assigned to a species or genus with similarity score between 90 and 99% (Table 2). Sequencing of the 16s *rRNA* gene revealed that *L. fermentum* was the prevalent species in smokers 11/12 (91.67%) while in non-smokers it represented 2/11(18.18%) of samples (Figure 1). However, in the non-smokers participants, *L. salivarius* was detected in 36.6 % of samples. *V. parvula* was the prevalent strain (100%) in non-smokers samples, while *V. tobetsuensis* was detected in 10/12 (83.3%) of smokers’ samples (Figure 1 and Supplementary Figure 3).

**TABLE 2:** Identification of isolated *Lactobacillus* spp. by 16s *rRNA* gene sequencing using BLASTn.

Identified species	Number of *Lactobacillus* isolates (Percent identity)
*Lactobacillus fermentum*	11 smokers and 2 non-smokers (95.21–96.79%)
*L. plantarum*	1 smoker and 2 non-smokers (95.54–96.23%)
*L. gasseri*	2 non-smokers (95.41%)
*L. salivarius*	4 non-smokers (95.57–95.62%)
*L. rhamnosus*	1 non-smoker (95.31%)

### Enumeration of *S. mutans, Lactobacillus* spp., and *Veillonella* spp. in Smokers and Non-smokers Groups

**FIGURE 2:**
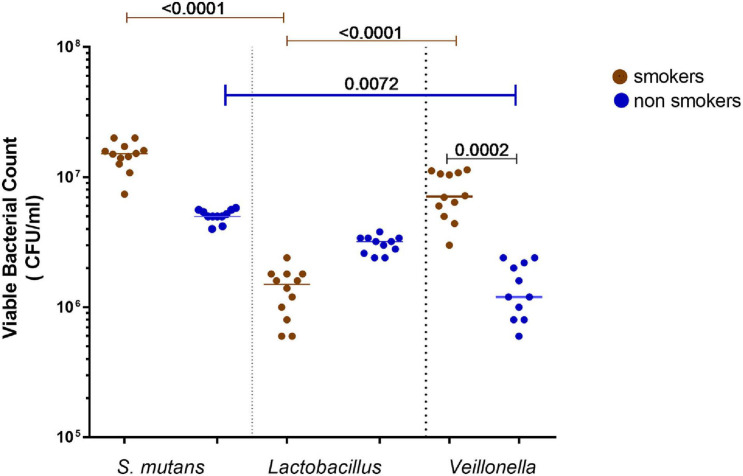
Total viable bacterial counts of isolated identified *Streptococcus mutans, Lactobacillus* spp., and *Veillonella* spp. from oral rinse of smokers (*n* = 12) and non- smokers (*n* = 11). Statistical differences calculated using Kruskal-Wallis followed by multiple comparisons using Dunn’s corrections. Data presented as median ± interquartile range, where *p* < 0.05 was considered to be statistically significant.

Results showed that *S. mutans* was the most prevalent bacteria in smokers’ samples, followed by *Veillonella* spp., then *Lactobacillus* spp. A significant increase (*P* < 0.001) between the viable counts of *Veillonella* spp. in smokers compared to non-smokers participants was detected. Among smokers, *S. mutans* showed higher viable counts than *Veillonella* spp. and *Lactobacillus* spp., respectively, while in non-smokers *S. mutans* viable counts was higher than *Lactobacillus* spp. and *Veillonella* spp., respectively (Figure 2).

### Determination of Anthracene Biodegradation by Oral Bacterial Isolates From Smokers and Non-smokers Measured by 2, 6-Dichlorophenol Indophenol (DCPIP) Assay

**FIGURE 3:**
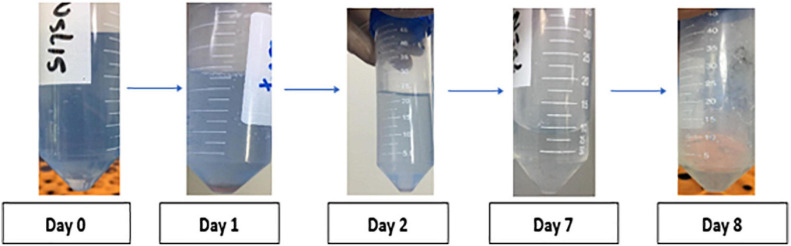
DCPIP assay of a representative smoker sample showing dye discoloration. during anthracene biodegradation over a period of 8 days in a representative smoker sample.

The results of preliminary estimation of anthracene biodegradation was determined by the ability of the bacterial isolates to reduce the blue color of DCPIP indicator in a period of 8 days. *V. parvula* being a highly reducer of blue color of DCPIP indicator after 8 days thus possessed the highest potential of anthracene biodegradation (Figure 3).

Culture dependent method of isolation made it impossible in some cases to compare results of anthracene degradation by the same species isolated from smokers and non-smoker group. This was mainly due to the fact that not all the species were isolated from both groups mainly due to depletion of some species in one group than the other. Results of anthracene biodegradation by individual bacterial isolates from smokers and non-smokers using 2,6-DCPIP colorimetric assay, compared to relevant standard strains showed that *S. mutans* isolated from smokers had significantly higher anthracene biodegradation ability than that from non-smokers and *S. mutans* ATCC 25175 at days 7 and 8 (*p* < 0.0001) (Supplementary Figure 4A). Anthracene biodegradation of *V. parvula* isolated from non-smokers was significantly higher than *Veillonella* spp. isolated from smokers, starting from day 1 to the end of the assay at day 8 (*p* < 0.0001). Moreover, *V. parvula* isolated from non-smokers showed significant differences in anthracene biodegradation than *V. parvula* ATCC 10790 and *V. dispar* ATCC 17748 (*p* < 0.0001) except at days 7 and 8 where no significant difference between *V. parvula* from non-smokers and *V. parvula* ATCC 10790 (Supplementary Figure 4B). For *Lactobacillus* spp. isolated from smokers and non-smokers, the results showed that at day 1, the anthracene biodegradation ability of smoker’s isolates was significantly higher (*p* = 0.0384) than non-smokers isolates, while at day 8, the ability of non-smokers isolates to degrade anthracene was significantly higher than isolates from smokers and *L. salivarius* ATCC 11741 (*p* = 0.0062) (Supplementary Figure 4C).

**FIGURE 4:**
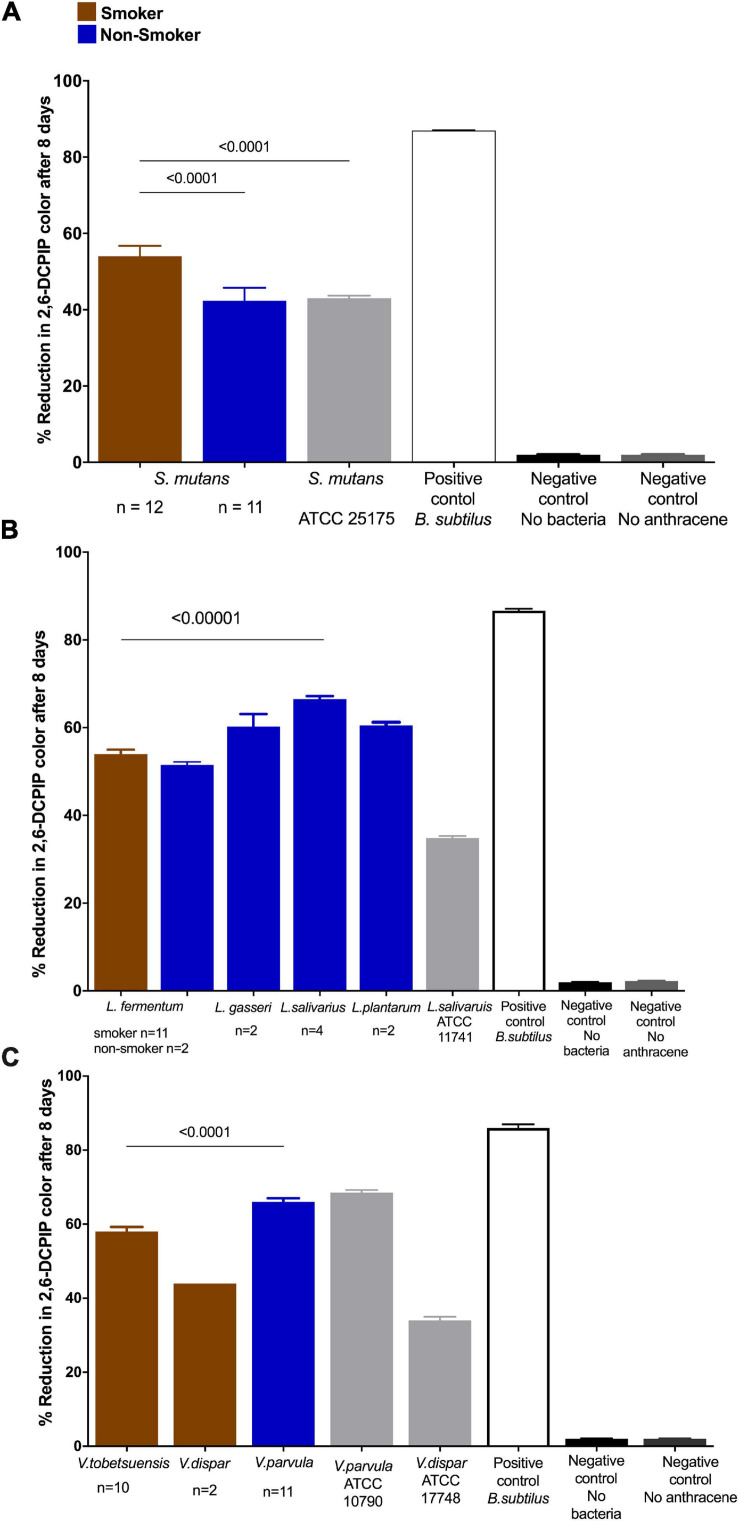
Anthracene biodegradation after 8 days in smokers and non-smokers compared to standard strains, positive and negative controls. Biodegradation of anthracene by **(A)** *S. mutans;* **(B)** *Lactobacillus* spp.; and **(C)** *Veillonella* spp. represented as color intensity (%) of DCPIP. Statistical differences calculated using Mann-Whitney *U*-test followed by multiple comparisons using Holm Sidak’s corrections. Data presented as median ± interquartile range, where *p* < 0.05 was considered to be statistically significant. *Bacillus subtilis* (positive control one of the highest anthracene biodegrading microorganisms) and negative controls (no anthracene or no bacteria) were significantly different from the isolated species and ATCC strains. ****p < 0.00001.

From previous results, the differences in anthracene biodegradation capability of bacterial species isolated from smokers and non-smokers was significant at the 8th day of reaction. Therefore, next comparison of data was represented as DCPIP color intensity % after 8 days of incubation using the 2, 6- DCPIP assay. *V. parvula* of non-smokers showed relatively higher anthracene biodegradation (median = 66.1%) than other isolates, while *V. dispar* of smokers (Figures 4A,C) showed relatively low ability to degrade anthracene (median = 44%) (Figure 4C). Non-smokers isolates of *S. mutans* (median = 44%) showed lower anthracene biodegradation ability than *V. parvula*, *L. gasseri*, *L. salivarius*, *L. rhamnosus*, and *L. plantarum.* For smokers’ subjects, the degradation potential of anthracene was 58, 53.7% for *L. plantarum* and *L. fermentum*, respectively, which is higher than *S. mutans* (median = 54.25%). Results of comparison of anthracene degradation by 2,6-DCPIP assay by individual bacterial isolates from smokers and non-smokers showed that *S. mutans* isolated from smokers (median = 54.25%) had higher anthracene degradation ability than that from non-smokers (Figure 4A). *Lactobacillus* spp. isolated from non-smokers including *L. gasseri, L. salivarius*, and *L. plantarum*, showed higher anthracene degradation potential than *L. fermentum* isolated from smokers’ samples (Figure 4B).

**FIGURE 5:**
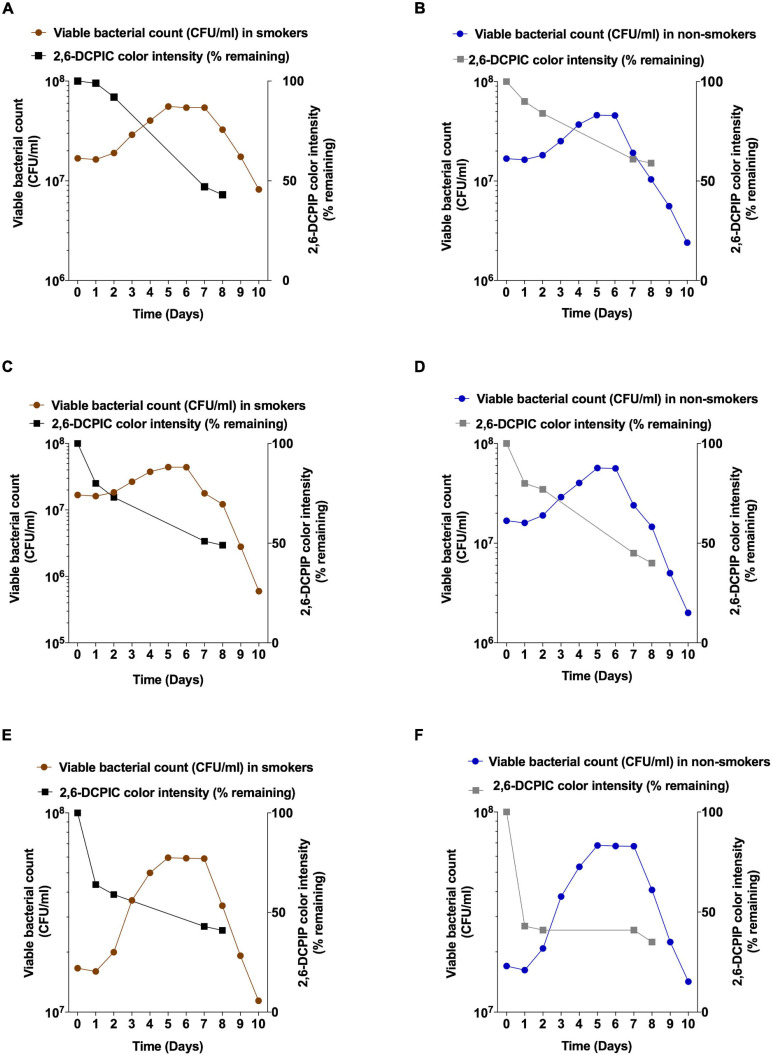
Viable counts and anthracene biodegradation by selected bacteria isolated from representative smoker and non-smoker participant over a period of 10 days. *S. mutans from* **(A)** smokers and **(B)** non-smokers **(C)** *L. fermentum* from smoker; **(D)** *L. salivarius* from non-smoker; **(E)** *V. tobetsuensis* from smoker; **(F)** *V. parvula* from non-smoker.

Next, we sought to assess the growth and anthracene degradation by various bacteria isolated from non-smokers and smokers. These bacteria were different as for instance, *V. parvula* was only isolated from non-smokers while it was depleted in smokers according to our results and in accordance with previous studies (Dalié et al., 2010; Wu et al., 2016). Changes in isolates viable counts during anthracene biodegradation were measured during a time of incubation of 10 days in a representative smoker participant and another from non-smoker participant to check the pattern of bacterial growth during the use of anthracene as a sole source of carbon and to confirm that anthracene biodegradation is reflected by an increase in bacterial viability. Acclimatized *S. mutans* with higher anthracene degradation showed higher growth than non-acclimatized *S. mutans* isolated from non-smokers (Figures 5A,B). *L. salivarius* showed higher level than *L. fermentum* in anthracene biodegradation potential which is reflected as higher viable bacterial counts (Figures 5C,D). *V. parvula* isolated from non-smoker participant showed higher degradation than *V. tobetsuensis* in the time period corresponding to lag and log phase of bacterial growth but after 8 days the degradation was nearly equivalent to each other. *V. parvula* showed higher viable counts than *V. tobetusensis* in the stationary phase (Figures 5E,F).

### Effect of Microbe-Microbe Interaction on Anthracene Biodegradation and Viable Bacterial Counts During DCPIP Assay

**FIGURE 6:**
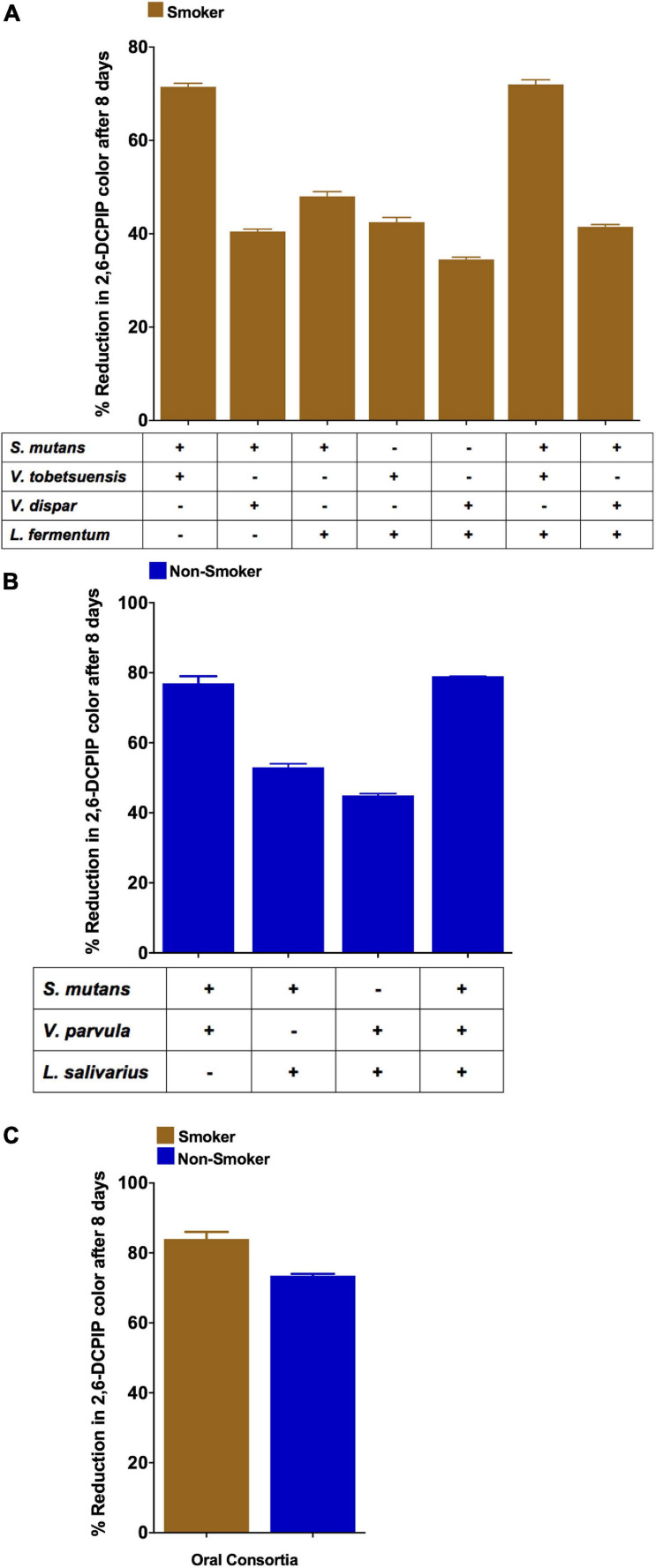
Anthracene biodegradation represented as DCPIP color intensity % by *S. mutans*, *Lactobacillus* spp. and *Veillonella* spp. mixtures after 8 days in smokers and non-smokers. **(A)** Duos and triples mixtures from smokers; **(B)** Duos and triples mixtures from non-smokers; **(C)** Consortium from representative smokers and non-smokers.

**FIGURE 7:**
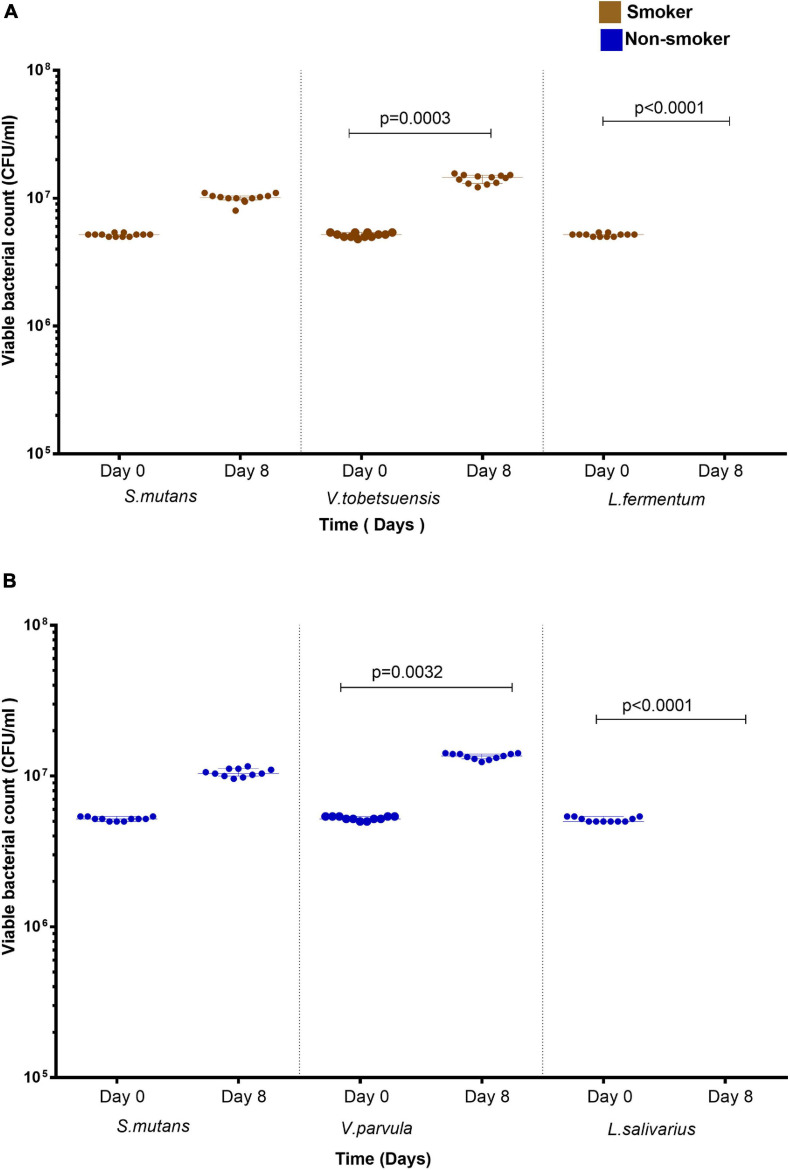
Viable bacterial count of *S. mutans*, *Lactobacillus* spp., and *Veillonella* spp. triple assay in presence of anthracene as a sole carbon source at day 0 and day 8. **(A)** smokers and **(B)** non-smokers. For *Lactobacillus* spp., no colonies was detected at day 8. Statistical differences calculated using Kruskal-Wallis followed by multiple comparisons using Dunn’s corrections. Data presented as median ± interquartile range, where *p* < 0.05 was considered to be statistically significant.

Co-cultures of duo and triple mixtures containing *V. dispar* showed lower biodegradation potential compared to other mixtures (Figure 6A). Duo mixtures containing *L.* salivarius with either *S. mutans* or *Veillonella* spp. showed lower biodegradation potential than mixtures containing *S. mutans* and *Veillonella* spp. in both smokers and non-smokers groups (Figures 6A,B and Supplementary Figures 5A–C). In triple mixtures, it was found that no viable cells were detected for *Lactobacillus* spp. after 8 days of incubation in presence of anthracene as sole source of carbon, and on the other hand *Veillonella* spp. viable counts were increased significantly (*P* < 0.001) after 8 days of incubation under the same conditions (Figures 7A,B). In consortium assay, the results showed that there was a significant increase in anthracene biodegradation ability by rinses from smokers compared to non-smokers starting from day 1 to the end of the assay at day 8 (Figure 6C and Supplementary Figure 5E).

## Discussion

Many factors such as smoking may affect both the composition and the function of oral microbiota. Cigarette smoke is one of the causes of dysbiosis, i.e. microbial imbalance or maladaptation of oral microbiota. This alteration in microbial profile by cigarette smoke could be attributed to different factors including direct contact with extensive microbes in the cigarette itself leading to acquisition and colonization by pathogenic bacteria. The oral microbial ecology may be also altered by cigarette smoke due to the direct effect with its released xenobiotics and toxicants on oral microbiota (Talhout et al., 2011). Other deleterious effects of cigarette smoking include suppression of immunity, increase of bacterial biofilm formation, depletion of local oxygen, decrease of salivary flow rate as well as lowering of pH of the oral cavity (Huang and Shi, 2019).

Cigarette smokers inhale many toxicants including more than 500 different polycyclic aromatic hydrocarbons (PAHs) compounds being identified in cigarette smoke (Hecht, 2011; Lawal, 2017). Polycyclic aromatic hydrocarbons, including anthracene, are classified as a major class of environmental genotoxins (Lawal, 2017). Screening of literature indicated a shortage of study and outcomes for the biodegradation of anthracene as an important member of PAHs by oral isolates. Therefore, this study can be considered innovative for comparing anthracene biodegradation by oral bacterial isolates from smokers and non-smokers, namely *S. mutans, Veillonella* spp., and *Lactobacillus* spp. Wu et al. (2016) suggested that cigarette smoking may affect the capability of oral microbiota to degrade PAHs, where abundances of PAHs biodegradation pathways were significantly altered in current smokers compared to never smokers. Therefore, the current study aimed to understand the mechanism by which selected bacterial members of oral microbiota are affected by exposure to cigarette smoking. The selected bacteria were *S. mutans*, *Veillonella* spp., and *Lactobacillus* spp. belonging to the most dominant oral phylum Firmicutes that represents 33% of the total oral microbiota. The selected bacteria were studied in regard to their counts and their ability to degrade one of the smoking hazardous compounds namely PAHs represented by anthracene using culture-dependent approach. Cigarette smoking was found to alter the relative abundance of the studied bacteria.

Enumeration of characteristic colonies of each genus on its selective medium revealed an increase in the prevalence of *S. mutans* in smokers compared to non-smokers. Such result as published by Wu et al. (2019), assumed a close relationship between the components of cigarettes particularly nicotine and the growth of cariogenic bacteria such as *S. mutans*. Cigarette smoking has been associated with an increased prevalence of dental caries where heavy smokers have significantly higher number of decayed, missing and filled teeth (DMFT) (Vellappally et al., 2007). *S. mutans* contributes to caries formation through acid production which damage dental enamel. The ability of *S. mutans* to synthesize the exopolysaccharides glucan and fructan by the glucosyl transferase (*gtfs)* and fructosyl transferases (*ftf)* genes, respectively, enables the bacteria-teeth binding and hence plaque formation (Wasfi et al., 2018). It was proved that nicotine significantly enhances biofilm formation and metabolic activities of *S. mutans* even at low concentration (Huang et al., 2012). In our study, *S. mutans* adapted to cigarette toxicant, as anthracene, isolated from smokers oral rinse showed higher biodegradation potential than those isolated from non-smokers and that was reflected in higher prevalence of this species in smoker samples. The reason behind the significant anthracene biodegradation potential of *S. mutans* in smokers compared to non-smokers could be explained with the theory of microbial adaption to xenobiotics. *S. mutans* isolated from anthracene polluted environment were found to be among high anthracene degradation genera (Karale et al., 2015). Microorganisms could adapt to the presence of xenobiotics by different mechanisms including :random mutations, increasing the resistance to toxic xenobiotics, mutations that enhance the ability of bacteria to degrade xenobiotics, acquiring genes coding for catabolic enzymes from other microorganisms by plasmids, transposons, integrons or phages (Haiser and Turnbaugh, 2013). All the previously mentioned mechanisms could explain the reason for the high abundance of *S. mutans* in smokers compared to non-smokers.

Concerning the genus *Lactobacillus*, no significant difference was detected between its salivary counts in smokers and non-smokers in agreement with a study done by Wu et al. (2016), but at the species level prevalence was significantly different. The prevalence of salivary *L. fermentum* was higher in smokers comprising about 92% of collected isolates compared to non-smokers (18.1%). In contrast to the high anthracene biodegradation pattern of *S. mutans* isolated from smokers, a substantial increase in biodegradation was observed with isolates from non-smokers’ samples rather than smokers’ ones. The low prevalence of high biodegrader *Lactobacillus* spp. in smokers may results from the rapid saturation of the enzymes responsible for anthracene biodegradation by anthracene itself in isolates recovered from smokers as mentioned by Wu et al. (2016). Therefore, the high prevalence of *L. fermentum* in smokers could be explained by the presence of a fully operational glutathione producing system in this species that removes reactive oxygen species (ROS) from the oral environment thus protecting the bacteria from the oxidative effect of cigarette smoking (Sardaro et al., 2019). The glutathione system allows this species to directly detoxify and eliminate ROS as hydrogen peroxide, hydroxyl radicals and lipid peroxyl radicals giving it a maximum protection against oxidative stress in smokers’ oral environment. The variation in the ability of different *Lactobacillus* spp. to reduce PAHs among different species as seen in the current study is conforming with the results of Yousefi et al. (2018). Different lactic acid producing bacteria have the ability to reduce xenobiotics and PAHs by two different mechanisms. The first one is by detoxification to less toxic metabolites which occurs when the presence of PAHs induces the production of aromatic ring deoxygenase enzyme known as one of the most common PAHs degrader enzymes (Abou-Arab et al., 2015). The second mechanism is by adsorption of these toxicants on the cell wall through physical interactions. It has been reported that the anionic functional groups present in the peptidoglycan layer, teichoic acids and teichuronic acids of gram positive bacteria are responsible for the bacterial toxic compound’s adsorption capacity (Yousefi et al., 2018) and that the proteins and carbohydrates present in the cell wall of lactic acid bacteria have an important role in toxins adsorption (Dalié et al., 2010).

As for the genus *Veillonella*, the study indicated a significant abundance of this genus in smokers compared to non-smokers, which is a result also verified by Wu et al. (2016) and Vallès et al. (2018). This finding suggests that cigarette smoking might have an impact on the physiology of the oral cavity creating an environment that favors the growth of strict anaerobic bacteria such as *Veillonella* spp. This is not surprising because one of the potential mechanisms by which cigarette smoking alters the oral microbial ecology is by oxygen depletion (Kilian et al., 2016; Yu et al., 2017; Huang and Shi, 2019). PAHs biodegradation by *Veillonella* spp. was not previously studied, but Dixit (2015) has mentioned *Veillonella* among the major anaerobic bacterial genera that are capable of degrading xenobiotics compounds. Gibson and Harwood (2002) reported that aromatic compounds such as PAHs can serve as electron acceptors to allow the anaerobic bacteria to transfer electrons to these compounds during anaerobic biodegradation process.

Identification of *Veillonella* to the species level showed that there was a significant increase in prevalence of *V. dispar* in smokers compared to non-smokers in accordance with the study of Moon et al. (2015) who found that *V. dispar* is one of the seven species occupying > 1% of the whole oral subgingival microbiota of smokers. PAHs biodegradation potential was not correlated with the ability of the *Veillonella* spp. to tolerate smoking stress in oral environment and this was concluded from the prevalence pattern of different *Veillonella* spp. in smokers and non-smokers samples. Comparing the biodegradation capability of *Veillonella* spp. isolates, a significant high anthracene biodegradation ability by *V. parvula* from non-smokers’ samples as compared to *V. tobetsuensis* and *V. dispar* isolated from smokers. The high PAH biodegradation potential by *V. parvula* may be the reason for the depletion of this species in smokers by rapid saturation of the degradation enzymes with toxic PAH leading to its death. It is also possible that the depletion of *V. parvula* is directly due to specific toxicant in cigarette smoke (Wu et al., 2016). A transcriptomic study of *V. dispar* and *V. parvula*, isolated from oral samples, showed a significant up-regulation of *V. dispar* genes which encode for oxidoreductase enzymes involved in the use of NAD^+^ and NADP^+^ as electron acceptors when compared to *V. parvula*, this might explain the increase in the prevalence of *V. dispar* in smokers due to the resistance of this species to the increase in ROS resulting from cigarette smoking by its oxidoreductase enzymes (Do et al., 2015). *V. dispar* was associated with few cases of infective endocarditis (Hillier et al., 2011) and the prevalence of this species in smokers might be one of the factors that enhance smokers’ associated increased risk of infective endocarditis (Strom et al., 2000; Huang and Shi, 2019).

The effect of microbe-microbe interactions on anthracene biodegradation ability along with its influence on viable bacterial count was studied. We tested several combinations of the oral isolates and the combinations giving the highest biodegradation potential with highest viable cells number were the co-cultures of *S. mutans* and *Veillonella* spp. except *V. dispar*. These results of anthracene biodegradation in mixture could be explained by the high biodegradation potential of *S. mutans* and *Veillonella* spp. in single cultures among collected isolates while *V. dispar* showed the lowest degradation potential (35%). The increased number of *S. mutans* and *Veillonella* spp. in co-culture was noticed by Kara et al. (2006) and Mashima and Nakazawa (2014) and when they found that the thickness of biofilms formed by *S. mutans*, in the presence of all the six oral *Veillonella* spp. was significantly higher than those formed of single *Streptococcus* spp. culture except for the mixture of *S. mutans* and *V. dispar* which is characterized by reduced biofilm formation by *S. mutans*. They suggested that the difference in mechanisms of biofilm formation, communication and coaggregation between the oral *Streptococcus* spp. and *Veillonella* spp. differs according to the species in mixed culture. Several studies reported that genera of *Streptococcus* and *Veillonella* are among the members of resident oral microbiota and they are both early colonizers of dental biofilm (Egland et al., 2004; Palmer et al., 2006; Liu et al., 2011, 2019; Mashima and Nakazawa, 2015).

Liu et al. (2011) mentioned that *V. parvula* is considered as a “friend” to *S. mutans* as they have been frequently detected in association in dental caries. Communities of both genera are considered as metabolic and co-aggregation partners in the human oral biofilm and that *Veillonella* spp. are not capable to colonize the dental surface without the aid of *Streptococcus* spp. during the early stages of oral biofilm formation (Palmer et al., 2006). The mutual relationship between *Streptococcus* spp. and *Veillonella* spp. is based on mutual benefits between the two genera where *Veillonella* spp. utilize short chains organic acids such as lactate produced by *Streptococcus* spp. for growth because *Veillonella* spp. is non-saccharolytic which make this genus unable to catabolize glucose or other sugars and rely on the fermentation of lactate and other organic acids to propionic and acetic acids (Mashima and Nakazawa, 2015). The lactate utilization by *Veillonella* spp. accelerates the glycolytic rate of *Streptococcus* spp. due to the removal of the lactate end product. *Veillonella* spp. also generate diffusible signal leading to upregulation of *Streptococcus* spp. amylase gene (*amyB*) which in turn increases the production of fermentable glucose and generates more lactate favoring the conditions for growth of *Veillonella* (Egland et al., 2004). *Veillonella* spp. relieve the microenvironment acidic pressure promoting the growth of *S. mutans* and resulting in balance between acid production and acid consumption, eventually leads to a constant pH, moreover it may lead to a less cariogenic environment *in vivo*. Microorganisms could adapt to the presence of xenobiotics by many mechanisms mentioned earlier, which may explain the increase in anthracene biodegradation ability by the consortium of smokers compared to that of non-smokers (Haiser and Turnbaugh, 2013).

In several studies, *Lactobacillus* spp. were found to play a beneficial role in inhibiting the growth of some cariogenic bacteria such as *S. mutans* (del Carmen Ahumada et al., 2001; Sookkhee et al., 2001; Simark-Mattsson et al., 2007; Badet and Thebaud, 2008; Rossoni et al., 2018). *L. casei*, *L. plantarum*, *L. rhamnosus*, *L. salivarius, L. fermentum*, and *L. paracasei* were found to exert the greatest antimicrobial activity against *S. mutans* (Koll-Klais et al., 2005; Teanpaisan et al., 2011). *L. fermentum* supernatant revealed the presence of antimicrobial compounds mainly organic acids, hydrogen peroxides and bacteriocins (Rodrigues et al., 2020). Phosphoric acid was produced by *L. fermentum* and it has strong antimicrobial properties against *S. mutans* (Prado et al., 2015), the bacteriocin detected in culture supernatant showed a broad spectrum of antimicrobial activity against gram positive bacteria such as *S. mutans* by formation of pores in the cytoplasmic membrane resulting in energy loss and alteration in the activity of important enzymes in the target bacteria (Mokoena, 2017). *L. fermentum* and its culture supernatant have the ability to decrease *S. mutans* adherence by inhibiting the production of water insoluble glucan by *S. mutans*, in addition to the production of biosurfactants which interfere with the adhesion of *S. mutans* to surfaces (Salehi et al., 2011). Overnight exposure of *S. mutans* to *Lactobacillus* spp. spent culture supernatant (SCT) showed an overall significant reduction in the expression of genes involved in virulence of *S. mutans* in planktonic and biofilm forming cells such as *atpD* and *aguD* genes which are acid tolerance genes helping *S. mutans* to tolerate acidity of the plaque environment and survival under acidic conditions, so downregulation of these genes can lead to bacteriostasis and eventual death (Wasfi et al., 2018).

## Conclusion

Cigarette smoking related oral dysbiosis was not limited to changes in oral bacterial counts but it extended to bacterial anthracene biodegradation tested in this study. Smoking participants’ oral rinse contained higher levels of viable *S. mutans, V. tobetsuensis*, and *V. dispar* and less of *V. parvula* compared to non-smoking participants. Although there was no difference observed in the total prevalence of *Lactobacillus* spp. between smokers and non-smokers; *L. fermentum* was significantly higher in smokers compared to non-smokers which might be due to the presence of fully operational glutathione system helping the bacteria to resist the oxidative stress resulting from cigarette smoking. Cigarette smoking affected *S. mutans* not only by changing its viable counts but also its anthracene biodegradation ability. *Streptococcus mutans* from smokers had higher anthracene biodegradation than non-smokers. Microbe –microbe interaction could account for changes in the anthracene biodegradation potential and the viable count of the microbial mixture compared to their corresponding single isolates and these changes differ according to the constituting genera. Singlets of *S. mutans* and *Veillonella* spp. showed higher anthracene biodegradation potential and viable counts than their co-cultures with *Lactobacillus* spp., suggesting that microbe-microbe interaction also affected anthracene biodegradation ability and not cigarettes toxicants only. The highest anthracene biodegradation ability was observed in the mixture of *V. parvula* with *S. mutans* indicating synergistic effect occurring in their co-culture.

## Data Availability Statement

The original contributions presented in the study are included in the article/Supplementary Material, further inquiries can be directed to the corresponding author/s.

## Ethics Statement

The studies involving human participants were reviewed and approved by the Research Ethics Committees (REC) of faculty of Pharmacy in October University for Modern Sciences and Arts (MSA) and Cairo University. The patients/participants provided their written informed consent to participate in this study.

## Author Contributions

SM, NA, and RW conceptualized the work. HM performed the experiments. SM, NA, RW, and HM analyzed the results. All authors edited the manuscript and contributed to the article and approved the submitted version.

## Conflict of Interest

The authors declare that the research was conducted in the absence of any commercial or financial relationships that could be construed as a potential conflict of interest.
