# Supplementary material for: High Counts and Anthracene Degradation Ability of Streptococcus mutans and Veillonella parvula Isolated From the Oral Cavity of Cigarette Smokers and Non-smokers
Source: Front Microbiol. 2021 Jun 28;12:661509. doi: 10.3389/fmicb.2021.661509 (PMC8273657; doi:10.3389/fmicb.2021.661509)
Supplement: Supplementary file 1 [file Data_Sheet_1.docx]

Supplementary Material

# Supplementary Figures and Tables

## Supplementary Figures

| 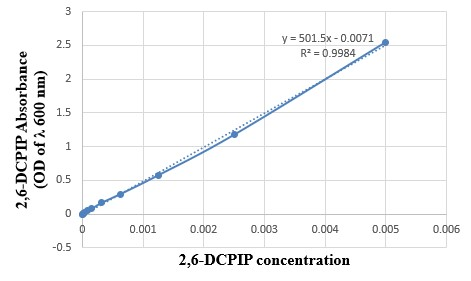 |
| --- |
| Supplementary Figure 1 Standard curve correlating the different concentrations of 2,6 DCPIP indicator with the corresponding absorbance at OD 600 nm.   \| 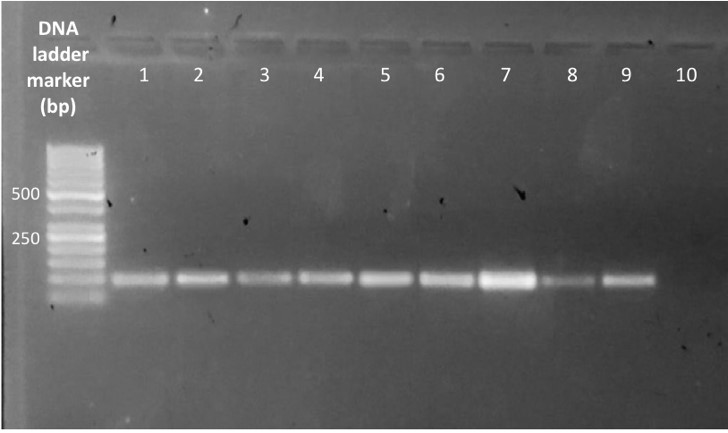  100 \| \| --- \| \| **Supplementary Figure 2.** Molecular identification of isolated *S. mutans* from selected smokers’ samples. PCR products obtained by amplifying *gtfb* gene fragment (96 bp). First lane: 50 bp DNA ladder marker; 1: *S. mutans* ATCC 25175; 2: SS1; 3: SS2; 4: SS3; 5: SS4; 6: SS5; 7: SS6; 8: SS7; 9: SS8, 10: negative control \|  \| A 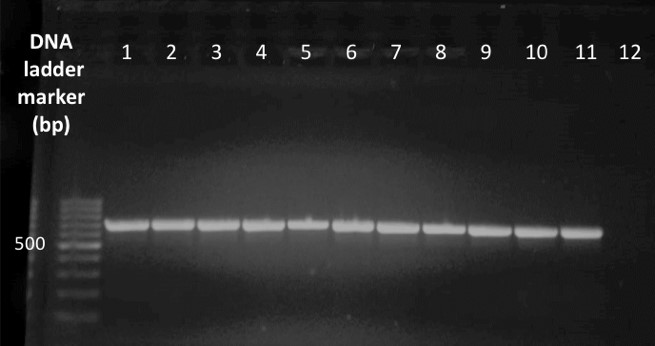 Amplicon size= 700 bp \| 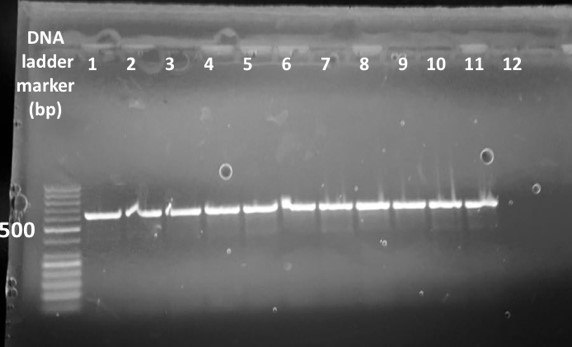B  Amplicon size= 623 bp bp \| \| --- \| --- \| \| 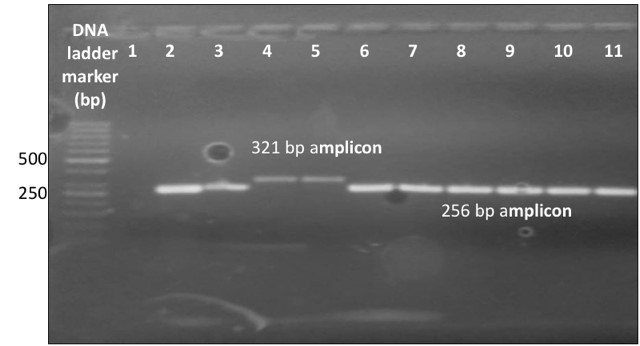  C \| \| \| **Supplementary Figure 3.** Molecular identification of *Veillonella* spp. from selected smokers and non-smokers’ samples. (A) Amplicons obtained by amplifying *Veillonella* spp*.* *rpoB* gene fragment 700 bp first lane 100 bp DNA ladder marker – Lane 1: *V. parvula* ATCC 10790; 2: SSV1; 3: SSV2; 4: SSV3; 5: SSV4; 6: SSV5; 7: SSV6; 8: SSV7; 9: SSV8; 10: SSV9; 11: SSV10; 12: negative control. (B) Amplicon of *rpoB* gene fragment differentiating *Veillonella parvula* (623 bp) in non-smokers’ samples; first lane: 50 bp DNA ladder marker; 1: *V. parvula* ATCC 10790; 2: NSV1; 3: NSV2; 4: NSV3; 5: NSV4; 6: NSV5; 7: NSV6; 8: NSV7; 9: NSV8; 10: NSV9; 11: NSV10; 12: negative control. (C) Amplicons of *rpoB* gene fragment *differentiating V. dispar* (321bp) and *dnak* gene fragment differentiating *V. tobetsuensis* (265 bp) in smokers’ samples*,* first lane: 50 bp marker; 1: negative control; 2: SV1; 3: SV2; 4: SV3; 5: SV4; 6: SV5; 7: SV6; 8: SV7; 9: SV8; 10: SV9; 11: SV10 \| \|  \| 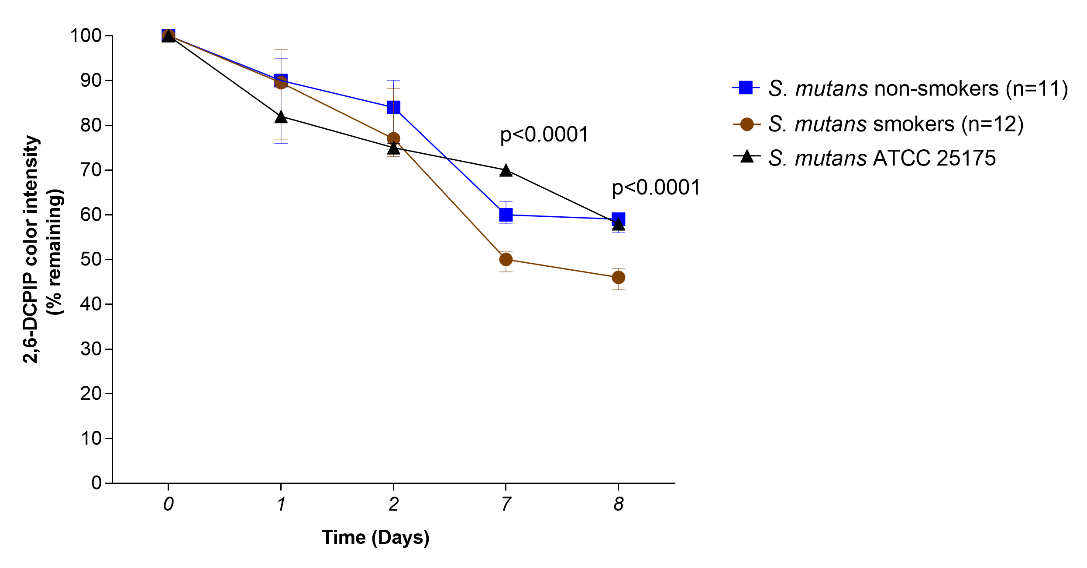  B  A \| \| --- \| \| 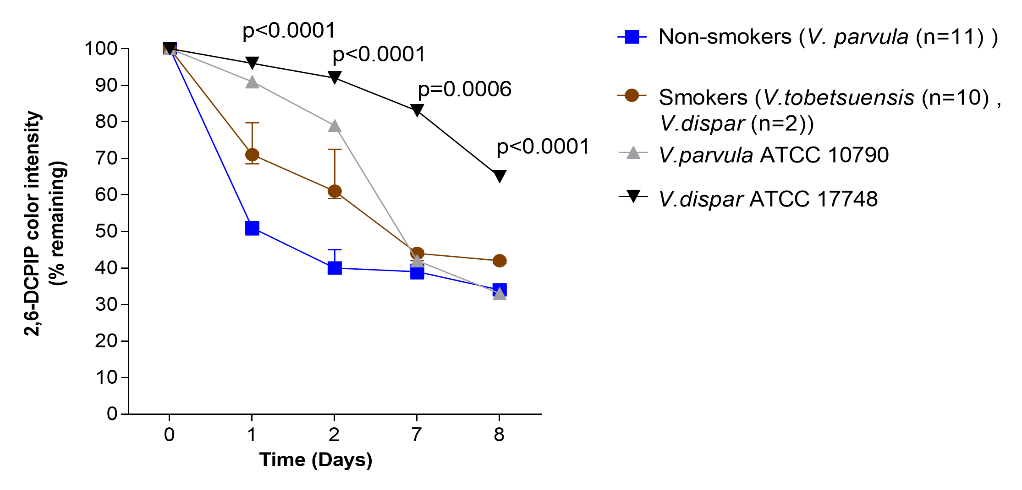  C \| \| 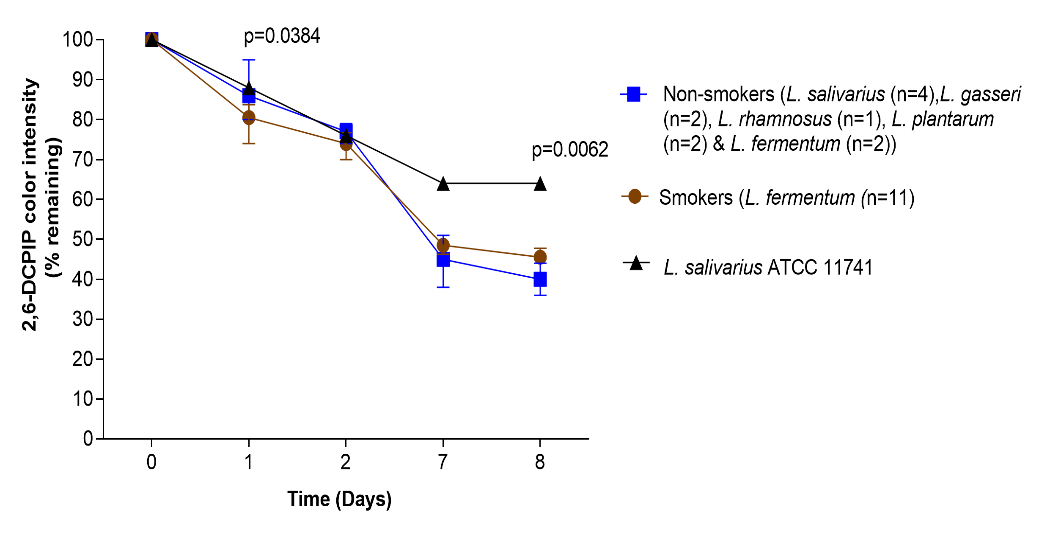 \|   **Supplementary Figure 4** Anthracene biodegradation of individual bacteria over a period of 8 days in smokers and non-smokers. Anthracene biodegradation by (A) *S. mutans,* (B) *Veillonella* spp., and (C) *Lactobacillus* spp. isolates represented as color intensity (%) of DCPIP   \| 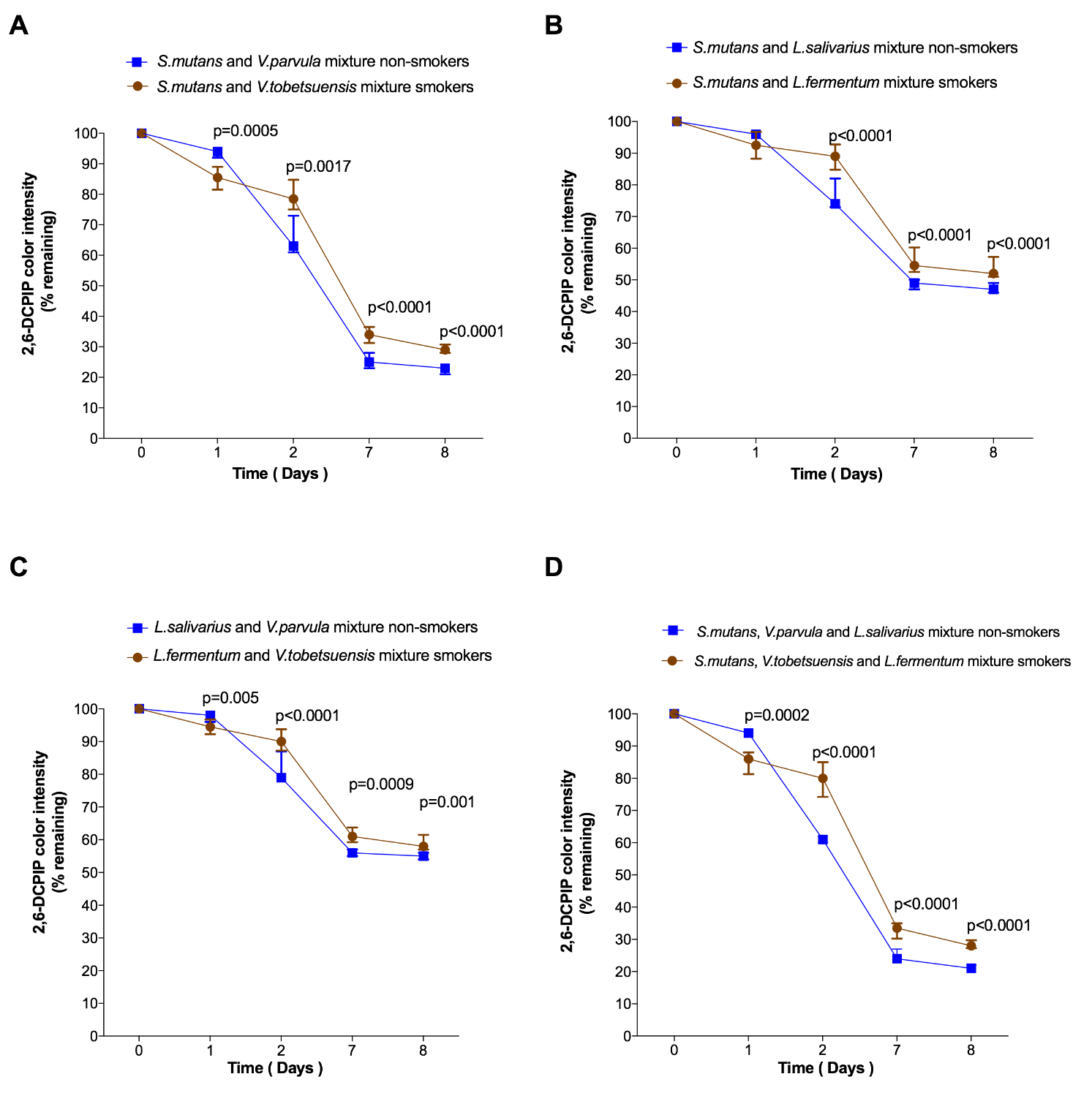 \| \| --- \|   **Supplementary Figure 5.** Estimated anthracene biodegradation efficiency of mixtures from smokers and non-smokers participants over a period of 8 days represented as reduction in DCPIP color intensity. (A) duo (1:1) mixture of *S. mutans* and *Veillonella* spp.; (B) duo (1:1) mixture of *S. mutans* and *Lactobacillus* spp.; (C) duo (1:1) mixture of *Lactobacillus* spp. and *Veillonella* spp.; (D) triple (1:1:1) mixture of *S*. *mutans*, *Lactobacillus* spp. and *Veillonella* spp.; (E) Consortium Supplementary Tables **Table (1): Demographic characteristics and smoking status of participants.**   \| Participants \| Smokers \| Non-smokers \| \| --- \| --- \| --- \| \| Number \| 14 \| 13 \| \| Gender (male/female) \| 13/1 \| 8/5 \| \| Average age (years) \| 21-40 \| 25-30 \| \| Average duration of smoking (years) \| 1-15 \| - \| \| Average cigarette’s consumption (Cigarettes/Day) \| 6-60 \| - \| |
